# Systematic review and meta analysis of differential attrition between active and control arms in randomized controlled trials of lifestyle interventions in chronic disease

**DOI:** 10.1186/s12874-021-01313-x

**Published:** 2021-06-14

**Authors:** Bevens W, Shoushtari A, Jelinek P, Jelinek GA, Weiland TJ

**Affiliations:** Centre for Epidemiology and Biostatistics, Melbourne School of Population and Global Health, The University of Melbourne, Melbourne, Australia

**Keywords:** Chronic disease, Attrition, Lifestyle, Retention

## Abstract

**Background:**

Attrition is a major obstacle for lifestyle interventions sustained for the medium-to-long term and can have significant consequences on the internal validity of a trial. When the degree of attrition differs between active and control arms this is termed differential attrition and is an important consideration during initial stages of trial planning.

**Objectives:**

The primary research question of this study was: what is the differential attrition between treatment arms in lifestyle interventions for prevalent chronic diseases?

**Methods:**

We performed a systematic review and meta-analysis of 23 studies involving a lifestyle intervention component in cohorts with chronic diseases. The search accessed three databases: Scopus, Medline Ovid and Web of Science. Attrition between treatment arms was analysed using a random-effects model and examined the relationship between the relative attrition and potential moderators, such as time to final follow-up, time to first follow-up, type of disease, type of control, type of intervention and length of treatment.

**Results:**

The pooled risk ratio was 1.00 (95% CI 0.97 – 1.03) and only one study fell outside this range. A univariable association was described between the pooled risk ration and length (years) to final follow-up, which did not remain in the multivariable model.

**Conclusions:**

Ultimately, we found no evidence of differential attrition in medium-to-long term lifestyle intervention studies for chronic disease, increasing confidence in conducting such studies with minimal potential of attrition bias.

**Trial registration:**

PROSPERO registration number CRD42018084495.

**Supplementary Information:**

The online version contains supplementary material available at 10.1186/s12874-021-01313-x.

## Background

Differential attrition (DA) is the difference in degree of loss to follow-up between the intervention and control arms of a trial. Attrition can introduce bias and affect the internal validity of a study’s findings [1]. Analysing DA may reveal useful information about factors that contribute to the engagement with new interventions [2].

The majority of meta-analyses pertinent to DA focus on specific clinical areas and have conflicting results. When medications are poorly tolerated, it stands to reason that dropout might be higher. In one large study published in the New England Journal of Medicine, 74% of patients discontinued the study medication before 18 months [3]. A wide-ranging difference in attrition was found in a meta-analysis examining first versus second generation antipsychotic drugs [4]. A smaller review of 10 trials investigating treatments for musculoskeletal disorders found a range of DA from 1–14% [5]. No DA was found in another two meta-analyses, one appraising type 2 diabetes monitoring^2^ and the other medication use for post-traumatic stress disorder [6].

A meta-analysis of attrition across a broad spectrum of clinical areas found no DA in randomised controlled trials (RCTs) [7]. Another study by the same group investigated DA in health behaviour change trials. They found slightly higher attrition in the intervention arm, however concluded that the differences were likely the result of sampling error [8].

To date, few studies have investigated DA in lifestyle intervention studies. The impact of attrition in such studies is largely unknown. There is a relative inability to achieve successful blinding in lifestyle interventions as participants are usually actively involved, meaning participants often know whether they have been randomized to the control or intervention arm. Understanding the causes of DA would improve the interpretation of trial results [9] and allow researchers to target factors that may increase retention.

This meta-analysis aims to determine the DA in RCTs of lifestyle interventions for common chronic diseases in Australia as published by the Australian Institute of Health and Welfare (AIHW) [10]. We were motivated to better understand whether any potential increased attrition due to a lack of engagement and incentive to continue in control arms might be potentially nullified by difficulties in motivation or adherence in intervention arms. This could better inform design of lifestyle intervention trials in the future.

## Methods

We conducted a systematic review and meta-analysis of RCTs in the following electronic databases prior to 23/3/2018: Scopus, Medline Ovid and Web of Science.

### Study population

Studies were selected based on a revised list of top chronic diseases affecting Australians according to the Australian Institute of Health and Welfare (AIHW). The chronic diseases that were considered for inclusion were: cardiovascular disease, chronic obstructive pulmonary disease (COPD), asthma, arthritis, type 2 diabetes and cancers with a 5-year survival rate of greater than 50%.

Studies to be included required interventions that were lifestyle oriented. The intervention must have contained at least one of: a diet, exercise, alcohol reduction, smoking cessation or stress management component. Studies where the primary intervention was vitamin supplementation were excluded as this intervention mimics closely adherence to pharmacological interventions.

Cancer studies that were considered for inclusion were limited to those which had a 5-year survival rate of greater than 50%. This was because certain cancers would fall outside of the term ‘chronic disease’ if mortality occurred close to diagnosis. Three researchers (PJ, WB and AS) then made the decision to split the sample into two groups, cancers and the remaining chronic diseases, and run these as separate meta-analyses, where results of the former will be published separately. The mechanisms of how study populations may respond to lifestyle interventions between the two groups was deemed to be different and therefore needed to be meta-analysed separately.

### Type of outcome measures

Studies were included if they reported the retention/attrition between baseline and follow-up. Retention was defined as the number of participants remaining in the study at each time point. Dropout/attrition was defined as the number of participants that refused participation at each time point.

### Search methods

The search procedure was conducted by the primary author with the assistance of an academic librarian. After running the initial search, a list of all articles was compiled and duplicates were deleted. Titles and abstracts of all remaining articles were screened by two independent reviewers (WB and AS). Irrelevant articles were discarded if the subject population did not have at least one chronic disease, the intervention fell outside the inclusion criteria, measurements were not taken or followed-up at ≥ 6 months, or the drop-out, retention, or attrition in the study were not reported.

### Search strategy

A systematic search was conducted on Scopus, Medline Ovid and Web of Science with the corresponding queries: [Abtract/title] *retention OR attrition OR dropout OR drop-out OR follow-up.*

*AND*

[Abtract/title] *diabetes OR asthma OR cardiovascular disease OR arthritis OR chronic obstructive pulmonary disease.*

*AND*

[Study type] *randomised control trial OR controlled trial OR RCT.*

### Selection of studies

Two reviewers (WB and AS) independently screened potentially eligible trials with disagreements resolved by a third author (PJ). WB and AS independently screened each study title and abstract for eligibility. The full texts of all remaining studies were examined for eligibility.

### Data Extraction

Data were extracted from the included papers independently by two reviewers (PJ and WB) using a form designed by the authors. This included: study population, country of study, length of treatment, type of intervention, length of first and last follow-up, type of control used, outcomes, remuneration, and number of participants in each arm at recruitment, beginning of the study period, and at final follow-up.

Study population was determined by the chronic disease identified within the paper; length of treatment was determined as the time over which an intervention was administered; type of control was coded as ‘wait-list’, ‘active control’ or ‘usual care’. Length of follow-up was divided into two categories: first follow-up and last-follow-up. Remuneration was indicated as either ‘yes’ or ‘no’, and the number of participants in each intervention and control arm at commencement of study period and at final follow-up were recorded.

### Quality assessment

The Revised Cochrane risk of bias tool for randomized control trials (ROB 2) was used to assess the quality of included trials [11]. Each study was assessed independently by reviews TW and WB according to screening questions with the 5 prescribed domains: risk of bias arising from the randomization process; risk of bias due to deviations from the intended interventions (effect of assignment to intervention); risk of bias due to missing outcome data; risk of bias in measurement of the outcome; risk of bias in selection of the reported result. Each screening questions was described assigned a low, medium or high score, and then an overall risk of bias score was calculated based on these domains. Any discrepancies were negotiated between TW and WB to be resolved by a third author if needed. Results are presented visually (Additional files 1 and 2).

### Analysis of studies

For each study, the proportion of participants lost to follow-up in both the intervention and control arms was calculated (*pI* and *pC*). The primary outcome of our meta-analysis was the risk ratio of pI/pC. A value > 1 indicated a higher degree of attrition in the intervention condition, and a value < 1 indicated a higher degree of attrition in the control condition.

The Stata (Stata version 15.1 was used) command *metan* was used to meta-analyse the studies. The input variables required by *metan* were contained in a 2 × 2 table (number of individuals who did and did not experience the ‘outcome event’ in either the treatment or control group). To compare the degree of attrition between the intervention group(s) and the control group, risk ratios were meta-analysed by the default Mantel–Haenszel method. Relative risk was computed separately for each study, weighted by their inverse variance and pooled to create a summary effect. Heterogeneity was assessed using the *I*^*2*^, tau^2^ and Cochran’s Q test, which are used to measure the percentage of between-study heterogeneity that is attributable to variability in the true treatment effect. Egger’s test with trim-and-fill was used alongside visual assessment of a funnel plot to determine publication bias.

Association between the pooled risk ratio and time to final follow-up, time to first follow-up, type of disease, type of control, type of intervention and length of treatment was assessed using univariable and multivariable meta regression models. Variables with only one observation were excluded from regression analysis, which was the case for cardiovascular disease, rheumatoid arthritis, wait list control.

## Results

**Fig. 1:**
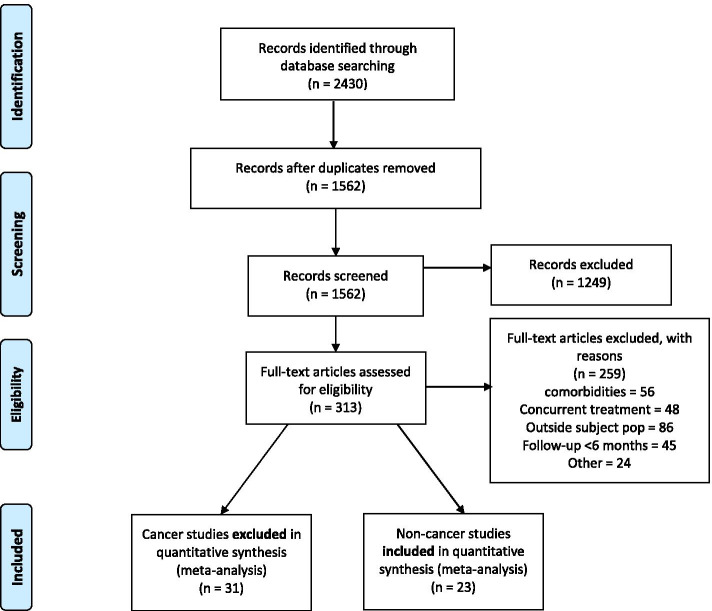
Prisma flow diagram

A total of 2430 studies were included following the literature search (Fig. 1). 1249 of those studies were removed following review of title and abstract by authors WB and AS. Subsequently, 313 articles were identified for full-text review. In all, 56 (21%) were excluded due to exhibiting co-morbidities, 48 (19%) were excluded due to concurrent treatment, 86 (33%) were excluded due to falling outside subject population, 46 (18%) were excluded due to a follow-up < 6 months, and 24 (9%) were excluded for other reasons. In this final review, 23 studies were included (Table 1).

**Table 1:** Study characteristics

Paper	Condition	Country	Length of treatment (months)	Type of Intervention	Length to initial follow-up (months)	Length to final follow-up (months)	Control
Khunti et al., 2012 [12]	Type 2 Diabetes	UK	0	Diet and Physical activity	36	36	Usual care
Hesselink et al., 2004 [13]	Asthma and COPD	Netherlands	Unclear	Counselling	12	24	Usual care
Andrews et al., 2011 [14]	Type 2 Diabetes	UK	12	Diet	6	12	Usual care
Clark et al., 2004 [15]	Type 2 Diabetes	UK	12	Diet	3	12	Usual care
Jonsdottir et al., 2015 [16]	COPD	Iceland	6	Smoking Cessation	6	12	Usual care
Lee et al., 2015 [17]	Type 2 Diabetes	Taiwan	3	Physical activity	3	12	Usual care
Meyer et al., 2015 [18]	Asthma	Germany	12	Physical activity	12	12	Usual care
Nicolucci et al., 2012 [19]	Type 2 Diabetes	Italy	3	Physical activity	12	12	Usual care
Walters et al., 2013 [20]	COPD	Australia	12	Smoking Cessation	6	12	Usual care
Wilson et al., 2008 [21]	COPD	UK	0	Counselling	12	12	Usual care
Barrera et al., 2006 [22]	Type 2 Diabetes	USA	6	Diet, physical activity, stress management	6	6	Usual care
Hilbernik et al., 2005 [23]	COPD	Netherlands	0	Smoking Cessation	6	6	Usual care
Nam et al., 2012 [24]	Type 2 Diabetes	USA	6	Physical activity	6	6	Usual care
Cheskin et al., 2008 [25]	Type 2 Diabetes	USA	8	Diet	20	20	Active control
Larsen, et al., 2011 [26]	Type 2 Diabetes	Australia	12	Diet	12	12	Active control
Praet et al., 2008 [27]	Type 2 Diabetes	Netherlands	12	Physical activity	12	12	Active control
Racine et al., 2011 [28]	Cardiovascular disease	USA	12	Diet	6	12	Active control
Callisaya et al., 2017 [29]	Type 2 Diabetes	Australia	6	Physical activity	6	6	Active control
Glasgow et al., 2000 [30]	Type 2 Diabetes	USA	6	Diet + Telephone support	3	6	Active control
Ng et al., 2011 [31]	COPD	Hong Kong	6	Physical activity	6	6	Active control
Sevick et al., 2012 [32]	Type 2 Diabetes	USA	6	Counselling	3	6	Active control
Yamada et al., 2014 [33]	Type 2 Diabetes	Japan	6	Diet	6	6	Active control
John et al., 2012 [34]	Rheumatoid arthritis	UK	1.8	CBT	6	6	Wait-list

In the 23 studies, the ratio of differential attrition ranged from 0.42 to 1.0 (41.9—100%) in the intervention and control conditions.

**Fig. 2:**
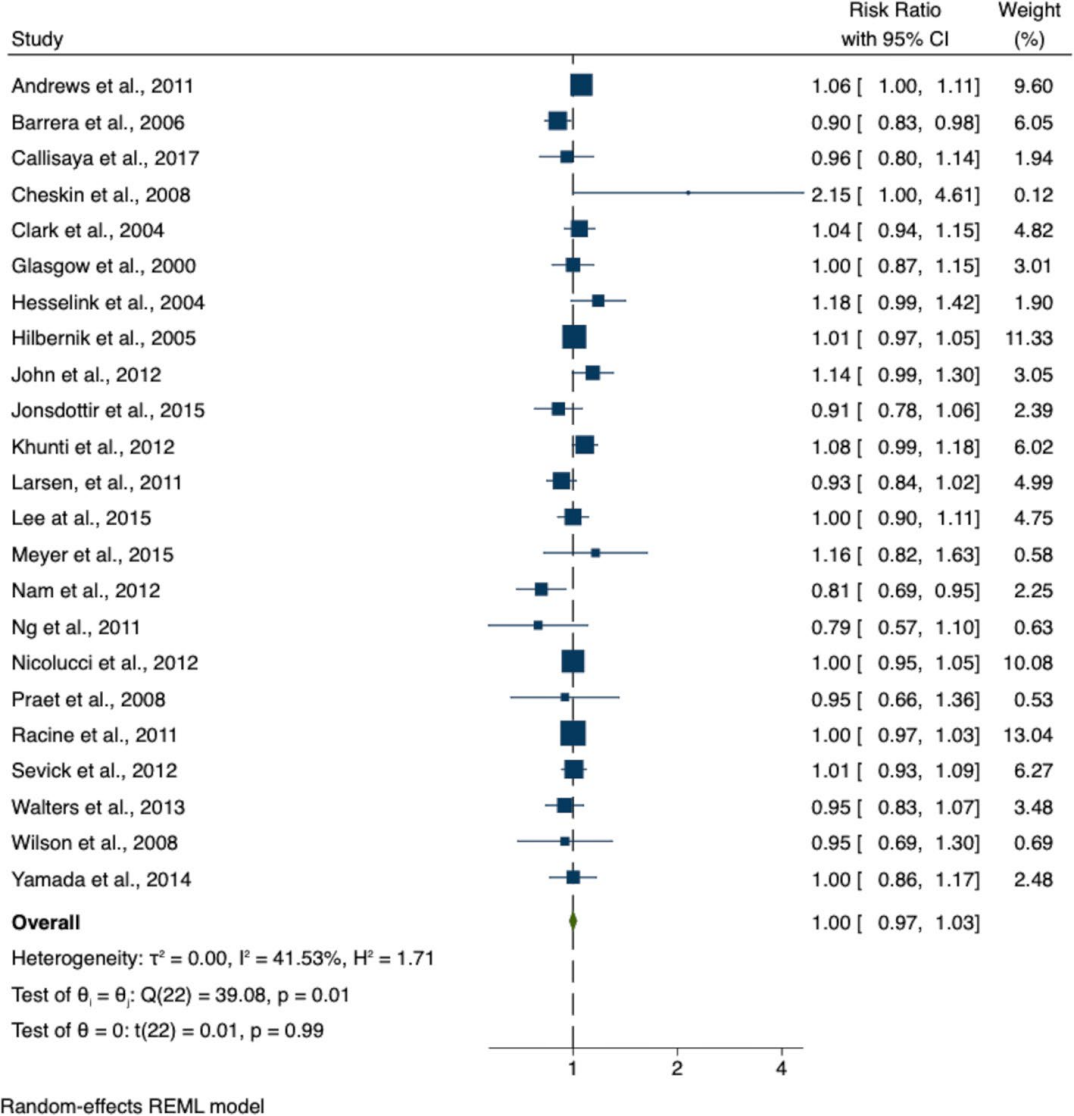
Forest plot for meta-analysis

The aim of this systematic review was to analyse the DA of lifestyle intervention studies, which may provide useful information about factors contributing to adopting these interventions. Table 2 displays the risk ratio of dropout in the control and intervention arms. Theta ranged from 0.79 (95% CI 0.57 – 1.101) to 2.15 (95% CI 1.00 – 4.61), with the pooled theta 1.00 (95% CI 0.97 – 1.03), which was not significant (Prob >|t|= 0.99). Two studies, Yamada et al., and Racine et al., had no loss to follow-up in either arm but were included via the method previously indicated. As shown in Fig. 2, one study (Nam et al.,) fell outside the pooled theta upper confidence interval.

**Table 2:** Meta-analysis summary table

Study	Risk ratio	95% CI	%weight
Racine et al., 2011 [28]	1.00	0.97 to 1.03	13.04
Yamada et al., 2014 [33]	1.00	0.86 to 1.17	2.48
Andrews et al., 2011 [14]	1.06	1.00 to 1.11	9.60
Barrera et al., 2006 [22]	0.90	0.83 to 0.98	6.05
Callisaya et al., 2017 [29]	0.96	0.80 to 1.15	1.94
Cheskin et al., 2008 [25]	2.15	1.00 to 4.61	0.12
Clark et al., 2004 [15]	1.04	0.95 to 1.15	4.82
Glasgow et al., 2000 [30]	1.00	0.87 to 1.15	3.01
Hesselink et al., 2004 [13]	1.18	0.99 to 1.42	1.90
Hilbernik et al., 2005 [23]	1.01	0.97 to 1.05	11.33
John et al., 2012 [34]	1.14	0.99 to 1.30	3.05
Jonsdottir et al., 2015 [16]	0.91	0.78 to 1.06	2.39
Khunti et al., 2012 [12]	1.08	0.99 to 1.18	6.02
Larsen, et al., 2011 [26]	0.93	0.84 to 1.02	4.99
Lee et al., 2015 [17]	1.00	0.90 to 1.11	4.75
Meyer et al., 2015 [18]	1.16	0.82 to 1.63	0.58
Nam et al., 2012 [24]	0.81	0.69 to 0.95	2.25
Ng et al., 2011 [31]	0.79	0.57 to 1.10	0.63
Nicolucci et al., 2012 [19]	1.00	0.95 to 1.05	10.08
Praet et al., 2008 [27]	0.95	0.66 to 1.36	0.53
Sevick et al., 2012 [32]	1.01	0.93 to 1.09	6.27
Walters et al., 2013 [20]	0.95	0.84 to 1.07	3.48
Wilson et al., 2008 [21]	0.96	0.69 to 1.30	0.69
exp(theta)	1.00	0.97 to 1.03

**Fig. 3:**
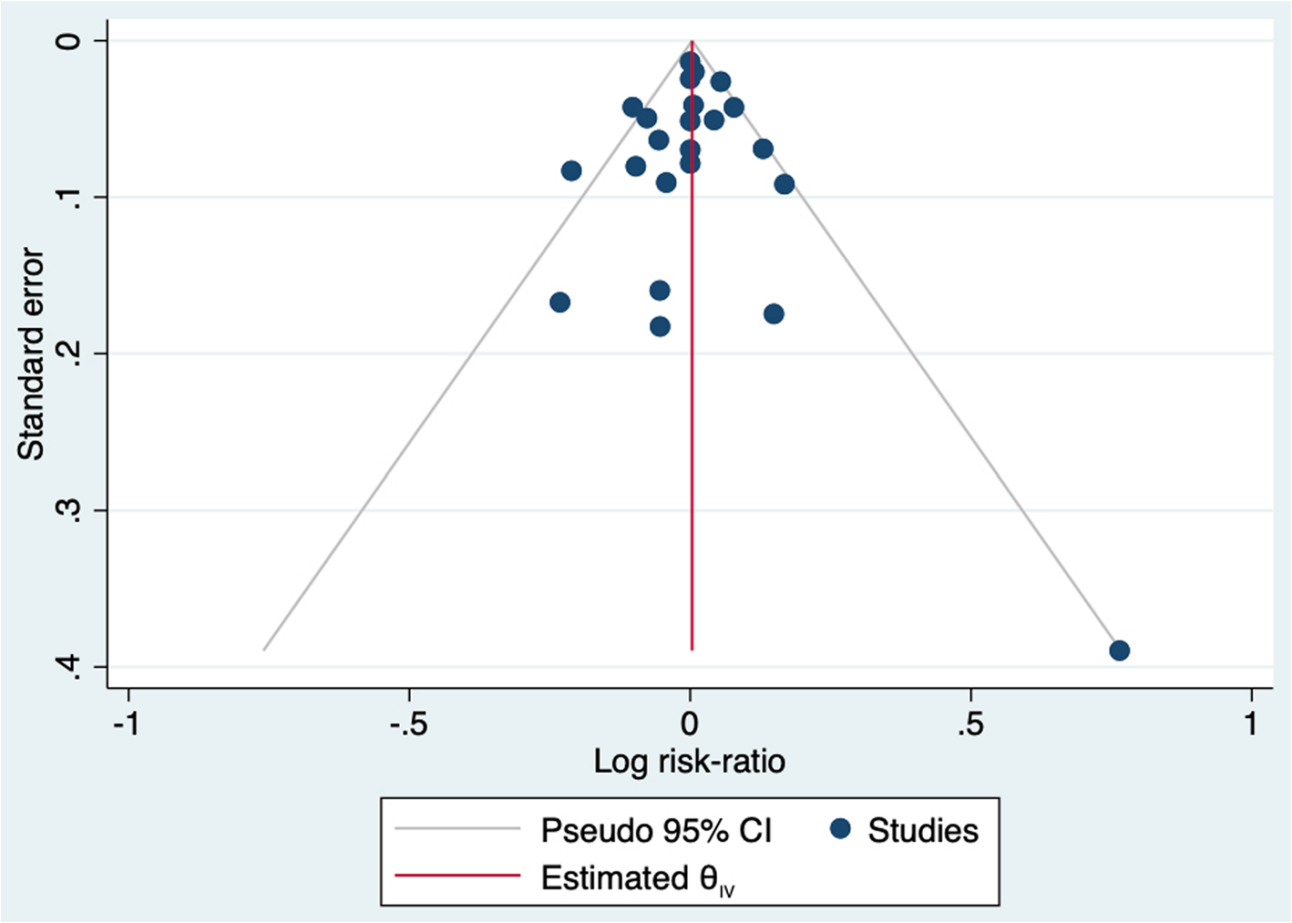
Funnel plot for publication bias

The funnel plot for the 23 studies included in the analysis is shown in Fig. 3. Neither the visual confirmation nor the Egger’s test for publication bias indicated asymmetry in the plot. Trim-and-fill method suggested one missing study on the mid-upper right side of the funnel plot, indicating a study with moderate attrition in the intervention arm. Imputation did not lead to any significant change in results, risk-ratio = 1.00 (95% CI 0.98 – 1.03).

Univariable meta regression described an association between time to final follow-up and pooled risk ratio (Table 3). This did not remain when adjusted for in the multivariable meta regression (Table 4). No other potential moderators described an association in either the univariable or multivariable models.

**Table 3:** Univariable associations

*Variable*	*Coefficient*	*95% CI*	*P*
Time to final follow-up (years)	0.05	0.012 to 0.094	0.03
Time to first follow-up (years)	0.03	-0.013 to 0.071	0.16
Length of treatment (years)	-0.02	-0.091 to 0.054	0.60
Active control	-0.02	-0.073 to 0.041	0.53
Wait-list control	0.13	-0.032 to 0.282	0.10
Self-report outcomes	-0.01	-0.071 to 0.044	0.68
Chronic obstructive pulmonary disease	-0.03	-0.101 to 0.048	0.49
Asthma	0.17	-0.006 to 0.343	0.06
Cardiovascular disease	0.01	-0.084 to 0.095	0.91
Rheumatoid Arthritis	0.14	-0.025 to 0.295	0.10
Physical activity	-0.05	-0.134 to 0.037	0.27
Cognitive behavioural therapy	0.05	-0.047 to 0.154	0.30
Smoking cessation	-0.04	-0.132 to 0.056	0.43
Multimodal	-0.02	-0.127 to 0.080	0.66

**Table 4:** Multivariable associations

		**Effect size, 95% confidence interval and *p-*value**	
*Variable*	*Coefficient*	*95% CI*	*P*
Time to final follow-up (years)	0.10	-0.076 to 0.275	0.27
Time to first follow-up (years)	-0.03	-0.208 to 0.156	0.78
Length of treatment (years)	-0.11	-0.259 to 0.032	0.13
Active control	-0.03	-0.150 to 0.081	0.56
Wait-list control	0.017	-0.256 to 0.290	0.90
Self-report outcomes	0.04	-0.070 to 0.152	0.47
Chronic obstructive pulmonary disease	-0.17	-0.427 to 0.089	0.20
Asthma	0.25	-0.130 to 0.633	0.20
Cardiovascular disease	-0.02	-0.175 to 0.130	0.77
Physical activity	-0.11	-0.228 to 0.017	0.09
Cognitive behavioural therapy	0.009	-0.158 to 0.175	0.92
Smoking cessation	0.05	-0.229 to 0.320	0.75
Multimodal	-0.18	-0.354 to 0.001	0.05

## Discussion

This meta-analysis demonstrates no DA in studies of lifestyle interventions for the chronic diseases investigated. The pooled theta of 1.00 indicates no difference in attrition between the intervention and control arms of included studies. There was no significant heterogeneity according to the *I* statistic with no significant publication bias findings, strengthening confidence in these results.

One of the 23 studies, Nam et al., fell outside the confidence intervals of the pooled estimate, with this study describing a higher degree of attrition in the intervention arm. The authors attributed this to the high “subject burden”, whereby participants were required to visit an exercise facility three times per week for 6 months. The mean total months of retention in the intervention arm was 2.7 months. Other studies using an exercise intervention, including those with type 2 diabetes as their subject population, used interventions that were not as intensive as Nam et al. This may explain why attrition in the intervention arm was not observed in those studies despite longer time to final follow-up and treatment length.

Meta regression described a univariable association between time to final follow-up and pooled risk ratio, which is intuitive. It might be expected that dropout would increase over time as participants lose motivation, interest, or find the longer follow-up burdensome. This association did not remain after adjusting for confounders in the multivariable model however, making it difficult to draw any meaningful conclusions. Interestingly, treatment length did not show a univariable association, nor did number of follow-ups. No other potential moderators were found to be associated.

The overall lack of DA in this meta-analysis may be an encouraging finding for future RCTs looking at lifestyle interventions in chronic conditions. If patients in future trials are similar to those in this review, they appear to be equally likely to stay in the study whether they are randomized to an intervention or a control arm, despite such studies having intuitive potential bias in attrition between arms. Conversely, it leaves little to analyse in terms of factors to target in order to increase retention. Future studies that report a difference in attrition should consider the factors for this difference and ways in which they could reduce DA.

### Limitations

The results of this study are limited to specific lifestyle interventions in the AIHW’s top 6 chronic diseases that affect Australians and therefore may not be applicable outside this specific cohort. Further, the majority (60%) of studies assessed in this meta-analysis were looking at patients with type 2 diabetes. This is not surprising as type 2 diabetes is an increasingly common chronic disease in western societies and receives a large amount of research attention. There is the possibility that studies with high differential attrition or high attrition in general are not submitted to journals or not accepted by journals, which might further bias these results. Further, the decision to not include cancer studies within the analysis, while appropriate, limits the power of our analysis and therefore, these results must be considered with caution. Additionally, records were not sourced from outside the listed databases and therefore other studies may have been missed. The strength of this study is the specificity of inclusion criteria and the modest sample size.

## Conclusion

Despite plausible hypotheses about the burden of lifestyle interventions in chronic disease treatment studies leading to increased attrition in one or other of treatment or control arms, we found no differential attrition at all between arms in a large group of studies of Australia’s most common chronic diseases. These results are encouraging but must be considered in light of the limited number of chronic diseases represented within this analysis. A future study should encompass a greater number of chronic diseases from a more diverse cohort.

## Supplementary Information

**Additional file 1.** Risk of bias figures; Visual representation of the summary of risk of bias assessment.

**Additional file 2.** Risk of bias graph; Visual representation of the risk of bias assessment combined for intention-to-treat and per-protocol.

## Data Availability

The datasets used and/or analysed during the current study are available from the corresponding author on reasonable request.

## Abbreviations

MS: Multiple Sclerosis
DA: Differential attrition
RCT: Randomised control trials
AIHW: Australian Institute of Health and Welfare
