# Supplementary figures and images for: Systematic review and meta analysis of differential attrition between active and control arms in randomized controlled trials of lifestyle interventions in chronic disease
Source: BMC Med Res Methodol. 2021 Jun 14;21:122. doi: 10.1186/s12874-021-01313-x (PMC8204467; doi:10.1186/s12874-021-01313-x)

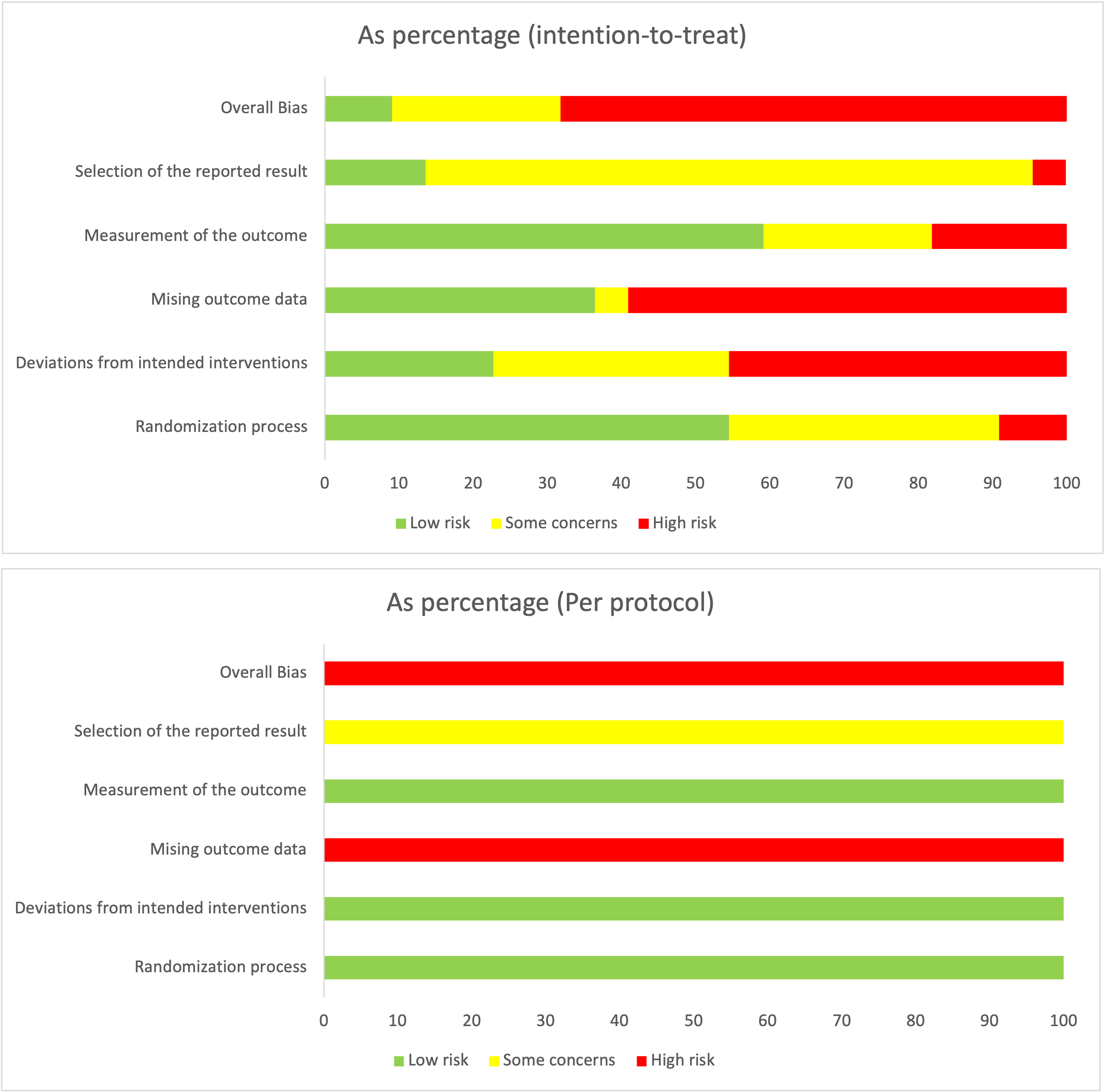

Supplement: Supplementary file 1 — Additional file 1. Risk of bias figures; Visual representation of the summary of risk of bias assessment. [file 12874_2021_1313_MOESM1_ESM.png]

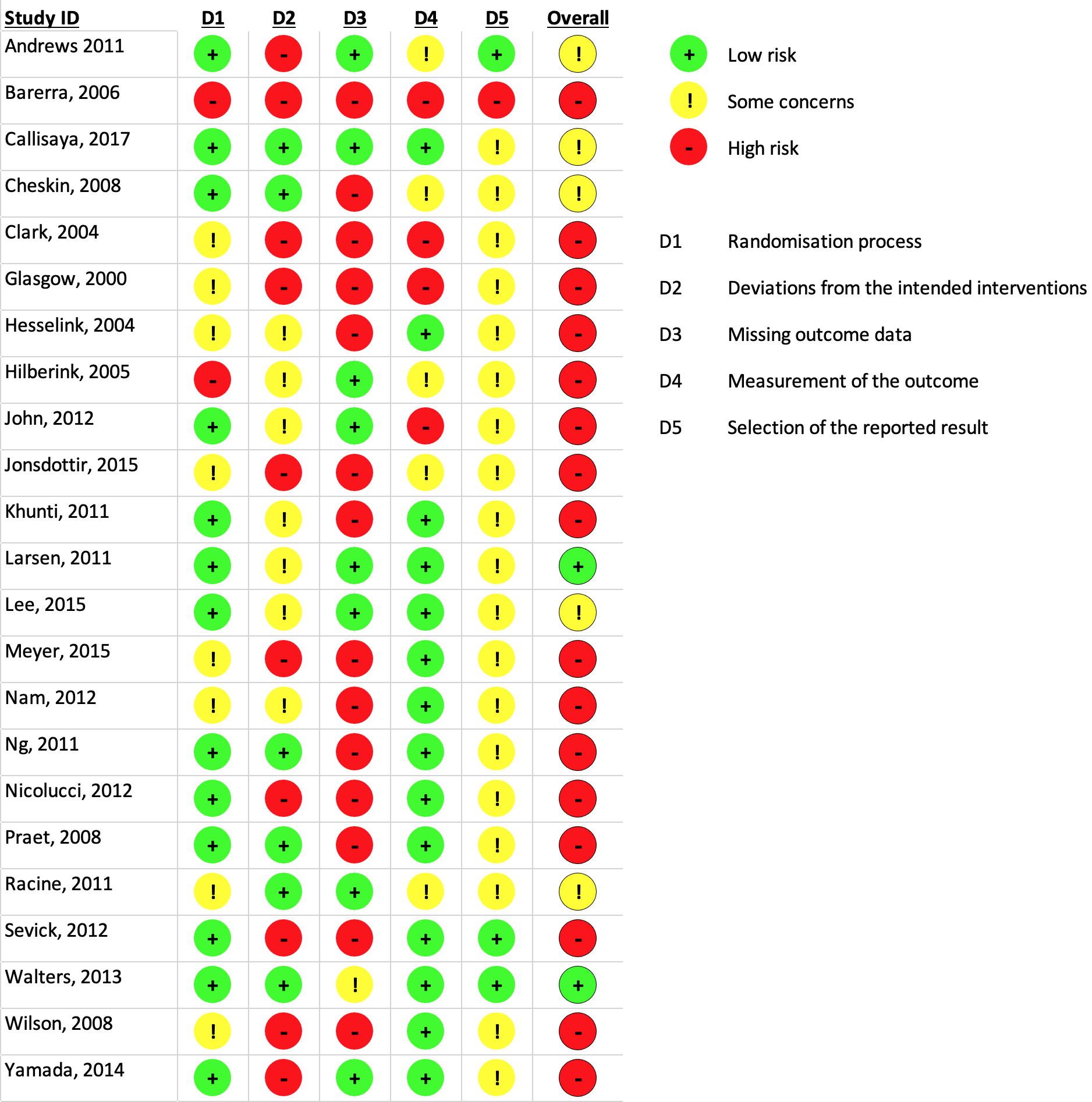

Supplement: Supplementary file 2 — Additional file 2. Risk of bias graph; Visual representation of the risk of bias assessment combined for intention-to-treat and per-protocol. [file 12874_2021_1313_MOESM2_ESM.png]
